# Clinical, genomic and immune microenvironmental determinants of nivolumab response in head and neck squamous cell carcinoma

**DOI:** 10.3389/fimmu.2024.1390873

**Published:** 2024-07-29

**Authors:** Takahiro Tsujikawa, Kazuchika Ohno, Kei-ichi Morita, Sumiyo Saburi, Junichi Mitsuda, Kanako Yoshimura, Alisa Kimura, Hiroki Morimoto, Hiroshi Ogi, Saya Shibata, Takumi Akashi, Morito Kurata, Issei Imoto, Yasushi Shimizu, Satoshi Kano, Akihito Watanabe, Tomoko Yamazaki, Yukinori Asada, Ryuichi Hayashi, Yuki Saito, Hiroyuki Ozawa, Kiyoaki Tsukahara, Nobuhiko Oridate, Daisuke Sano, Arata Horii, Yushi Ueki, Takashi Maruo, Nobuaki Mukoyama, Nobuhiro Hanai, Takahito Fukusumi, Hiroshi Iwai, Takuo Fujisawa, Takashi Fujii, Ken-ichi Nibu, Shigemichi Iwae, Tsutomu Ueda, Nobuyuki Chikuie, Ryuji Yasumatsu, Mioko Matsuo, Hirohito Umeno, Takeharu Ono, Muneyuki Masuda, Satoshi Toh, Kyoko Itoh, Shigeru Hirano, Takahiro Asakage

**Affiliations:** ^1^ Department of Otolaryngology-Head and Neck Surgery, Kyoto Prefectural University of Medicine, Kyoto, Japan; ^2^ Department of Cell, Developmental, and Cancer Biology, Oregon Health & Science University, Portland, OR, United States; ^3^ Department of Head and Neck Surgery, Tokyo Medical and Dental University, Tokyo, Japan; ^4^ Department of Maxillofacial Surgery, Tokyo Medical and Dental University, Tokyo, Japan; ^5^ Department of Pathology and Applied Neurobiology, Kyoto Prefectural University of Medicine, Kyoto, Japan; ^6^ SCREEN Holdings, Kyoto, Japan; ^7^ Department of Diagnostic Pathology, Tokyo Medical and Dental University, Tokyo, Japan; ^8^ Department of Comprehensive Pathology, Tokyo Medical and Dental University, Tokyo, Japan; ^9^ Aichi Cancer Center Research Institute, Nagoya, Japan; ^10^ Department of Medical Oncology, Faculty of Medicine and Graduate School of Medicine, Hokkaido University, Sapporo, Japan; ^11^ Department of Otolaryngology-Head and Neck Surgery, Faculty of Medicine and Graduate School of Medicine, Hokkaido University, Sapporo, Japan; ^12^ Department of Otolaryngology- Head and Neck Surgery, Keiyukai Sapporo Hospital, Sapporo, Japan; ^13^ Department Head and Neck Oncology Division, Saitama Medical University International Medical Center, Hidaka, Japan; ^14^ Department of Head and Neck Surgery, Miyagi Cancer Center, Natori, Japan; ^15^ Department of Head and Neck Surgery, National Cancer Center Hospital East, Kashiwa, Japan; ^16^ Department of Otolaryngology-Head and Neck Surgery, Graduate School of Medicine, The University of Tokyo, Tokyo, Japan; ^17^ Department of Otorhinolaryngology, Head and Neck Surgery, Keio University School of Medicine, Tokyo, Japan; ^18^ Department of Otorhinolaryngology, Head and Neck Surgery, Tokyo Medical University, Tokyo, Japan; ^19^ Department of Otorhinolaryngology, Head and Neck Surgery, Yokohama City University, School of Medicine, Yokohama, Japan; ^20^ Department of Otolaryngology Head and Neck Surgery, Niigata University Graduate School of Medical and Dental Sciences, Niigata, Japan; ^21^ Department of Otorhinolaryngology, Nagoya University Graduate School of Medicine, Nagoya, Japan; ^22^ Department of Head and Neck Surgery, Aichi Cancer Center Hospital, Nagoya, Japan; ^23^ Department of Otorhinolaryngology-Head and Neck Surgery, Osaka University Graduate School of Medicine, Osaka, Japan; ^24^ Department of Otolaryngology, Head and Neck Surgery, Kansai Medical University Hospital, Osaka, Japan; ^25^ Department of Head and Neck Surgery, Osaka International Cancer Institute, Osaka, Japan; ^26^ Department of Otolaryngology–Head and Neck Surgery, Kobe University Graduate School of Medicine, Kobe, Japan; ^27^ Department of Head and Neck Surgery, Hyogo Cancer Center, Akashi, Japan; ^28^ Department of Otolaryngology and Head and Neck Surgery, Hiroshima University Hospital, Hiroshima, Japan; ^29^ Department of Otorhinolaryngology-Head and Neck Surgery, Faculty of Medicine, Kindai University, Osaka, Japan; ^30^ Department of Otorhinolaryngology, Graduate School of Medical Sciences, Kyushu University, Fukuoka, Japan; ^31^ Department of Otolaryngology-Head and Neck Surgery, Kurume University School of Medicine, Kurume, Japan; ^32^ Department of Head and Neck Surgery, National Hospital Organization Kyushu Cancer Center, Fukuoka, Japan

**Keywords:** head and neck squamous cell carcinoma, nivolumab, biomarker, immune profile, mutation

## Abstract

**Background:**

In view of improving biomarkers predicting the efficacy of immunotherapy for head and neck squamous cell carcinoma (R/M HNSCC), this multicenter retrospective study aimed to identify clinical, tumor microenvironmental, and genomic factors that are related to therapeutic response to the anti- Programmed cell death protein 1 (PD-1) antibody, nivolumab, in patients with R/M HNSCC.

**Methods:**

The study compared 53 responders and 47 non-responders, analyzing formalin-fixed paraffin-embedded samples using 14-marker multiplex immunohistochemistry and targeted gene sequencing.

**Results:**

Of 100 patients included, responders had significantly lower smoking and alcohol index, higher incidence of immune related adverse events, and higher PD-1 ligand (PD-L1) expression in immune cells as well as PD-L1 combined positive score (CPS) than non-responders. The frequency of natural killer cells was associated with nivolumab response in patients with prior cetuximab use, but not in cetuximab-naïve status. Age-stratified analysis showed nivolumab response was linked to high CPS and lymphoid-inflamed profiles in patients aged ≥ 65. In contrast, lower NLR in peripheral blood counts was associated with response in patients aged < 65. Notably, *TP53* mutation-positive group had lower CPS and T cell densities, suggesting an immune-excluded microenvironment. Patients with altered tumor suppressor gene pathways, including *TP53*, *CDKN2A*, and *SMAD4* mutations, had lower CPS, higher smoking index, and were associated with poor responses.

**Conclusion:**

Nivolumab treatment efficacy in HNSCC is influenced by a combination of clinical factors, age, prior treatment, immune environmental characteristics, and gene mutation profiles.

## Introduction

Programmed cell death protein 1 (PD-1) antibody therapy has been widely used in real-world practice for recurrent and metastatic head and neck squamous cell carcinoma (R/M HNSCC) since the results of the CheckMate-141 trial, which investigated the efficacy and safety of nivolumab in platinum-resistant HNSCC (1). However, the low response rate to immunotherapy remains a clinical challenge (2), requiring the prediction of responders who would benefit from anti-PD-1 treatment. Finding predictive biomarkers for anti-PD-1 antibodies and improving the therapy sequence are both necessary to optimize the choices for treating R/M HNSCC.

Biomarkers that predict the therapeutic responses to immunotherapy have been widely explored by monitoring tissue-derived components (3–5). PD-1 ligand (PD-L1) expression in tumor and immune cells has been reported to correlate with the therapeutic effect of PD-1/PD-L1 inhibitors in head and neck cancer, and is currently the only U.S. Food and Drug Administration (FDA)-approved biomarker (6). However, high PD-L1 expression does not always correlate with a response because of potential background factors including tumor and tumor microenvironmental (TME) profiles; further biomarkers are needed (7). TME is a complex network of cells, molecules, and extracellular matrix that surrounds and interacts with tumor cells to modulate the immune response to the tumor and the efficacy of immunotherapy (8). Although it has been suggested that T-cell inflammatory signatures correlate with the efficacy of PD-1 therapy, little is known about how the intratumoral cellular composition and density of immune cells are related to therapeutic efficacy (9).

In addition to TME profiles, the genetic characteristics of cancer are closely related to the therapeutic effect of immunotherapy (10, 11). Tumor mutation burden (TMB) is a measure of the number of mutations in the tumor genome, which reflects the neoantigen load and immunogenicity of the tumor, and high TMB is associated with a favorable response to anti-PD-1 therapy in many types of cancer (12). However, HNSCC is known to have low correlations between TMB and intratumoral T cell frequency (12), which can compromise the accuracy of predicting the anti-PD-1 therapeutic effect based on TMB. Furthermore, the individual genetic mutations that are frequent in HNSCC, such as *TP53*, *PIK3CA*, and *NOTCH1* are not well understood for their impact on the immune environment. Together, these results indicate that integrated genomic and TME-based profiling is required to identify predictive biomarkers for immunotherapies in HNSCC.

The present study sought to identify biomarkers that predict the therapeutic response to the anti-PD-1 antibody, nivolumab, based on a multicenter retrospective study to select the best course of treatment for patients with R/M HNSCC. Here we have identified PD-L1 expression, intratumoral immune profiles, genomic alterations as tissue-derived biomarkers for nivolumab response in patients with R/M HNSCC. These biomarkers may aid in enhancing treatment selection and overcoming anti-PD-1 therapeutic resistance.

## Methods

### Clinical samples and clinicopathological information

This study included patients with R/M HNSCC who received nivolumab treatment across 22 facilities from March 1, 2017, to May 31, 2018. Out of a total of 613 cases, clinicopathological information and formalin-fixed, paraffin-embedded (FFPE) tissue sections were obtained from 53 responders and 47 non-responders as a case-control study (**Supplementary Figures 1A, B**). Patients who achieved complete or partial response according to RECIST version 1.1 were defined as responders, and patients with stable disease or progressive disease as their best overall response were defined as non-responders. Nivolumab was administered every two weeks until disease progression, occurrence of unacceptable adverse events, or death. Patients who had not been previously treated with platinum-containing chemotherapy, used nivolumab as adjuvant chemotherapy, or received nivolumab in combination with other antineoplastic agents were excluded. Age, sex, primary tumor site, progression, differentiation, smoking and alcohol consumption history, serum albumin level, C-reactive protein (CRP) level, lymphocyte count, neutrophil count, best overall response, immune-related adverse events, cycles of nivolumab use, prior cetuximab exposure history, and Eastern Cooperative Oncology Group (ECOG) performance status (PS) were obtained. The cut-off values of cell counts and ratios were determined according to previous reports (13, 14). Smoking history was evaluated using ta smoking index (pack/year) calculated as a number of packs smoked daily multiplied by number of years of smoking. Alcohol consumption history was evaluated using an alcohol consumption index calculated by multiplying the amount of pure alcohol/27 g per day by the number of years of drinking. Human papilloma virus (HPV) status was determined by institutional pathologists using p16 immunohistochemistry (IHC) for oropharyngeal cancers.

### Multiplex IHC

Multiplex IHC was performed with 4 or 5 μm of formalin-fixed, paraffin-embedded (FFPE) tissue sections as previously described (15). Briefly, FFPE tumor sections were subjected to sequential immunodetection using antibodies against immune cell lineages. Following the chromogen development of the antibodies, the slides were scanned digitally at a 20x objective magnification using a NanoZoomer S60 scanner (Hamamatsu Photonics). A complete list of antibodies and the conditions used for staining is provided in **Supplementary Table 1**.

### Digital image processing and image cytometry

Following staining, image acquisition and computational processing were performed as previously described (15). Iteratively digitized images were accurately aligned using in-house software (SCREEN Holdings Co., Ltd.). The software calculated the coordinates of each image relative to the reference image. A series of uncompressed TIFF images of each ROI was retrieved based on these coordinates. Visualization was performed using ImageScope Version 12.3.3.5048 and ImageJ. Co-registered images were converted to single-marker images, inverted, and converted to grayscale, followed by pseudo-coloring. Single-cell segmentation and staining intensity quantification were performed for quantitative image assessment, using CellProfiler Version 2.2.0. All pixel intensity and shape-size measurements were saved in a file format compatible with the image cytometry data analysis software, FCS Express 7 Image Cytometry Version 7.06.0015 (*De Novo* Software).

Tumor and immune cells expressing PD-L1 were quantified according to the lineage identification markers in **Supplementary Table 2**. Immune profiles were identified based on the criteria obtained from our previous study on HNSCC: hypo-inflamed group with less than 1500 immune cells per mm^2^, lymphoid-inflamed group with lymphoid immune cell to myeloid immune cell ratio of 2:1 or more, and myeloid-inflamed group with less than a 2:1 ratio (15).

### Sequencing analysis

A flowchart of the gene mutation validation process is shown in **Supplementary Figures 2A, B**. The GeneRead DNA FFPE Kit (Qiagen, Hilden, Germany) was used to extract gDNA from the FFPE tissues including uracil-DNA glycosylase treatment prior to amplification. After quality control exclusions, 62 cases were included for genomic DNA extraction and sequencing. AmpliSeq for the Illumina Comprehensive Cancer Panel (Illumina, California, USA), a target panel for examining the exon regions of 409 cancer-associated genes, was used to prepare the libraries. The prepared libraries were sequenced using the NextSeq 500/550 High Output Kit v2.5 (300 Cycles) (Illumina) on the next-generation sequencer NextSeq (Illumina), and fastq and bam data were obtained by Local Run Manager (Illumina). Hg19 was used for the alignment.

### Genomic data analysis

The obtained fastq data were analyzed for gene mutations using the supercomputer, SHIROKANE at the Institute of Medical Science, University of Tokyo, and the analysis pipeline, Genomon 2.6.3 (https://genomon-project.github.io/GenomonPagesR/). Germline mutations were removed on allele frequencies in public databases, i.e., the ToMMo 8.3KJPN-SNV/INDEL Allele Frequency Panel (jmorp.megabank.tohoku.ac.jp), the Human Genetic Variation Database (www.genome.med.kyoto-u.ac.jp/SnpDB/), the 1000 Genomes Project database (www.1000genomes.org), and the NHLBI GO Exome Sequencing Project (esp.gs.washington.edu). Additional review using Integrative Genomics Viewer (IGV, version 2.12.3, Broad Institute, Cambridge, MA, USA) identified and excluded low-mappability regions and sequencing errors. The VAF cutoff was 0.04 according to the previous report (16). Copy number analyses were performed using the Number Targeted Resequencing Analysis (CONTRA, sourceforge.net/projects/contra-cnv/) (17). TMB ≥ 10 was defined as TMB-high (TMB-H), and TMB < 10 as TMB-low (TMB-L).

### Statistics

Chi-square, and Kruskal-Wallis tests were used to compare the frequencies of categorical and continuous variables, respectively. Multivariate analysis was performed using multiple logistic regression analysis with JMP14.2.0 (SAS Institute Inc, Cary, NC, USA). Unsupervised hierarchical clustering was performed using Ward’s minimum variance method (hclust from R) in the R v. 4.3.0 software (http://www.r-project.org). Statistical calculations were performed using JMP14.2.0 (SAS Institute Inc., Cary, NC, USA) and GraphPad Prism 8.3.0. P values < 0.05 were considered statistically significant.

## Results

### Clinicopathological factors associated with a nivolumab response

**Figure 1:**
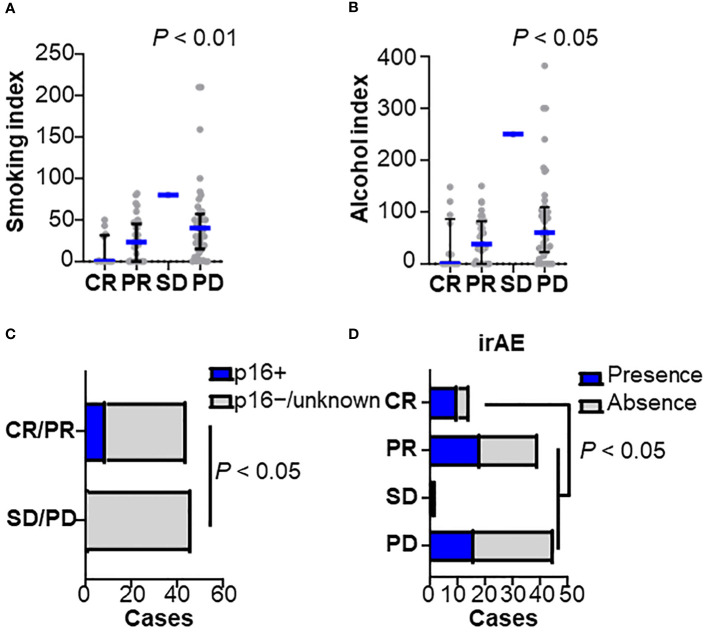
Clinicopathological characteristics related to nivolumab efficacy Baseline characteristics were stratified by the best overall responses to nivolumab in **(A)** smoking index (pack/year), **(B)** alcohol consumption index, **(C)** human papilloma-virus (HPV) status, and **(D)** presence of immune-related adverse events (irAEs) (CR, n = 14; PR, n = 39; SD, n = 2; PD, n = 45). Bars and whiskers represent median and interquartile ranges, respectively. Statistical differences were determined using the Kruskal-Wallis test.

Of the patients studied, 53 were responders (CR/PR) and 47 were non-responders (SD/PD). We examined sex, age, performance status, HPV status, smoking history, primary site, stage at diagnosis, prior cetuximab use, CRP level, lymphocyte count, and neutrophil-to-lymphocyte ratio (NLR) (**Figures 1A–D**). Univariate analysis showed that female sex, non-smoking, p16 positive, low CRP (0.6 ≤), and low NLR (< 7) were significantly associated with a nivolumab response (**Table 1**). Low smoking index was significantly associated with a therapeutic response, but not with HPV status (22.8 in HPV-positive versus 33.8 in HPV-negative cases on average smoking indexes) (**Figure 1A**). A similar trend was observed with indexed alcohol consumption (**Figure 1B**). HPV positivity was observed in 9% of all cases and significantly correlated with the nivolumab response (**Figure 1C**). Multivariate analysis revealed that smoking history were an independent predictor where non-smokers were more likely to be responders (**Table 1**). Among the post-treatment clinical factors, the presence of immune-related adverse events was associated with a treatment response (**Figure 1D**). Immune-related adverse events of grade 3 or higher occurred in 5 cases in the responder group, which included arthritis (G3), anemia (G3), adrenal insufficiency (G3), and two cases of hepatobiliary disorders (G3). In the non-responder group, there were 4 cases with adverse events of grade 3 or higher, which included two cases of enterocolitis (G3), one case of enterocolitis (G4), and pneumonitis (G3). A treatment response was not associated with the primary sites (**Supplementary Figure 1B**). Together, these observations indicate that the clinical factors associated with a nivolumab treatment response were the absence of smoking history, and the presence of immune-related adverse events, requiring further investigation of the biological background.

**Table 1:** Univariate and multivariate analysis for nivolumab responses.

	Univariate			Multivariate		
	Odds ratio	P value	95% CI	Odds ratio	P value	95% CI
Sex						
Male V.S. female	3.126	0.049*	1.005–11.973	2.180	0.331	0.452–12.244
Age						
< 65 V.S. 65 ≤	0.713	0.401	0.321–1.568			
Peformance states						
0-1 V.S. 2-4	0.410	0.227	0.097–1.741			
Smoking history						
Presence V.S. absence	8.444	0.0002***	2.584–38.318	5.964	0.009**	1.533–30.637
Stage						
I/II V.S. III/IV	0.596	0.380	0.171–1.874			
Prior cetuximab use						
Presence v.s. absence	1.530	0.2916	0.695–3,414			
p16 status						
Positive v.s. negative/unknown	0.106	0.008**	0.006–0.600	0.181	0.086	0.009–1.239
CRP						
< 0.6 V.S. ≥ 0.6	0.433	0.043*	0.188–0.975	0.456	0.138	0.157–1.288
ALC						
< 600 v.s. ≥ 600	0.907	0.846	0.331–2.433			
NLR						
<7v.s. ≥ 7	0.341	0.025*	0.124–0.876	0.371	0.109	0.101–1.245

ALC, absolute lymphocyte count; NLR, neutrophil-to-lymphocyte ratio; *< 0.05; **< 0.01; ***< 0.001.

### Immune microenvironmental factors associated with a nivolumab response

**Figure 2:**
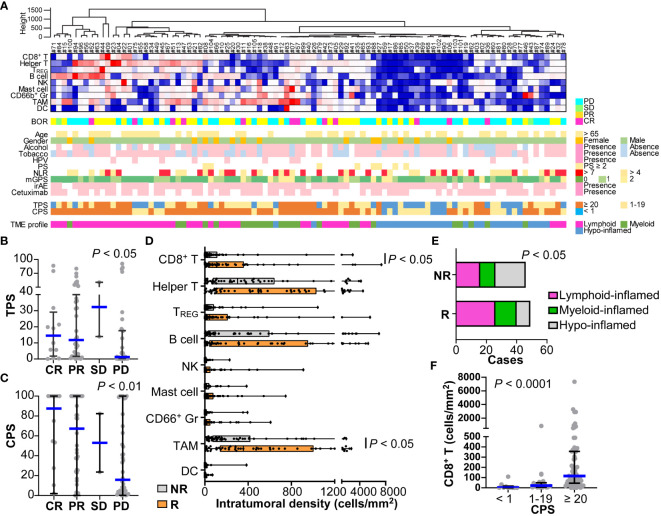
Immune characterization based on 14-marker multiplex IHC comparing responders and non-responders for nivolumab in HNSCC. **(A)** Intratumoral cell densities (cells/mm^2^) of nine immune cell lineages in each case were quantified using multiplex IHC and image cytometry. A heatmap according to the color scale (upper right) is shown with a dendrogram of unsupervised hierarchical clustering (n = 95). Best overall response (BOR), baseline characteristics, the presence of immune-related adverse events (irAE), prior cetuximab (Cmab) usage, and PD-L1 expression by tumor proportion score (TPS) or combined positive score (CPS) are shown below. Tumor microenvironmental (TME) profiles of lymphoid-, myeloid-, and hypo-inflamed groups were determined by the cut-off values according to the previous study (see Methods). **(B, C)** PD-L1 expression scores by TPS **(B)** and CPS **(C)** were shown, comparing different nivolumab responses. **(D)** Immune cell densities were quantified, comparing responders and non-responders. **(E)** Immune profiles comparing subgroups are shown. Statistical differences were determined via the Chi-square test. **(F)** CD8^+^ T cell densities comparing different nivolumab responses. Bars and boxes/whiskers represent the median and interquartile ranges, respectively in **(B–D, F)**. Statistical differences in **(B–D, F)** were determined via Kruskal-Wallis tests.

14-marker multiplex immunohistochemistry and image cytometry analysis were performed on FFPE tissue sections, and each single cell was compartmentalized on the image for flow cytometry-like quantification as previously described (15). Immune cell densities and compositions with PD-L1 expression were evaluated quantitatively (**Figure 2A** and **Supplementary Figure 3A**). Five of the 100 slides (#31, #33, #54, #76, and #96) were excluded due to glass slide incompatibility. In the analysis of the tumor proportion score (TPS), the percentage of tumor cells expressing PD-L1, TPS in the responder group were significantly higher than those of the non-responder group (**Figure 2B** and **Supplementary Figure 3B**). This trend was further pronounced in the combined positive score (CPS), where PD-L1 expression in both tumor and immune cells was considered. A significantly higher CPS was observed in the responder group than in the non-responder group (**Figure 2C** and **Supplementary Figure 3C**). The number of responses was particularly low (1 out of 7 cases, 14.4%) in patients with a CPS of less than 1, suggesting a negative predictive ability of negative CPS.

The compositions and cell densities of CD45^+^ immune cell lineages were comparatively analyzed in relation to the therapeutic response and clinicopathological factors (**Figures 2A, D**, **Supplementary Figure 3A**). The intratumoral immune cell frequency in responders tended to be higher than that in non-responders, where the percentage of immune cells in the total cells was significantly higher in responders than in non-responders (**Supplementary Figure 3D**). In particular, CD8^+^ T cells and macrophages had significantly higher cell densities in responders (**Figure 2D**). No correlation was observed between individual immune cell frequencies and smoking history or the NLR.

Clustering analysis based on immune cell densities revealed that clusters with low immune cell densities were associated with non-responders, whereas clusters with high immune cell densities tended to have favorable responses (**Figure 2A**). Next, we stratified the immune characteristics into three groups of hypo-, lymphoid-, and myeloid-inflamed groups based on criteria from our previous study, where the balance of antitumoral lymphoid versus immunosuppressive myeloid cells was related to prognosis in HNSCC (see Methods) (15). Both the lymphoid- and myeloid-inflamed groups showed a significantly higher frequency of responses than the hypo-inflamed group (**Figure 2E** and **Supplementary Figure 3E**). The CPS was significantly lower in the hypo-inflamed group, and positively correlated with CD8^+^ T cell density (**Figure 2F**), suggesting an association between high CPS and a favorable response. CD66b^+^ granulocyte/lymphocyte ratios in tumor tissues showed no significant correlation with NLR in the circulating blood or with other standard blood parameters, indicating the significance of analyzing the characteristics of the tissue, not only in the peripheral blood (**Supplementary Figure 3F**).

### Response-related immune characteristics and clinical factors stratified by treatment history and age

**Figure 3:**
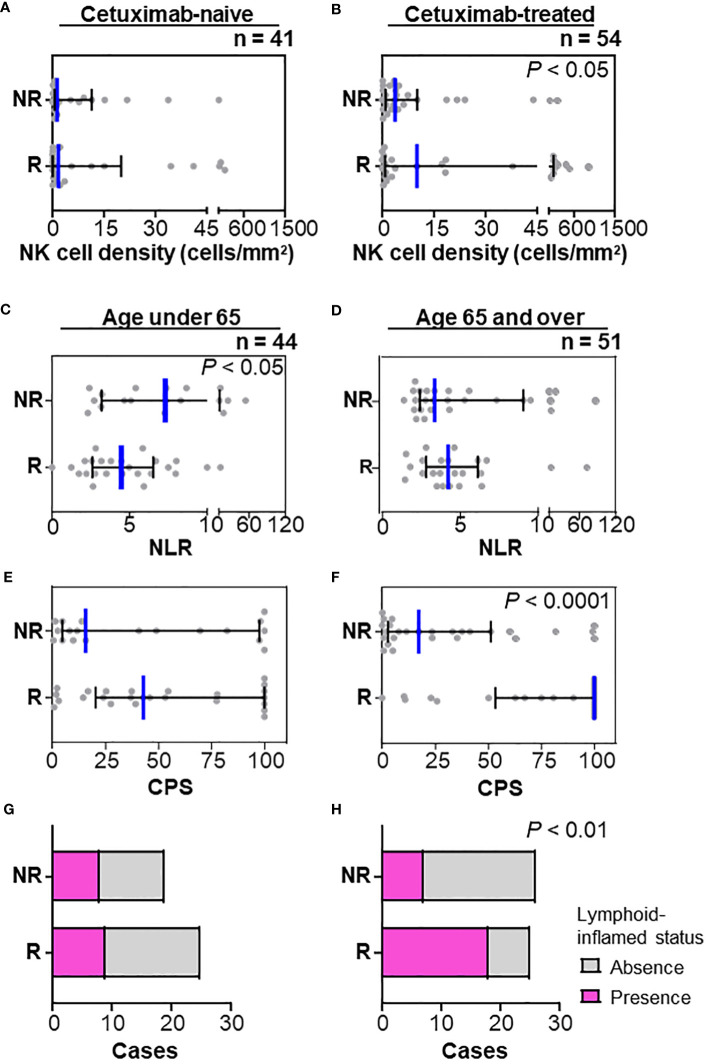
Stratification by prior cetuximab use and age in relation to the nivolumab response. **(A, B)** Stratification by the history of prior therapy revealed that the frequency of natural killer (NK) cells was associated with a nivolumab response in patients with prior cetuximab use **(A)** but not in a cetuximab-naïve status **(B)**. **(C, D)** Neutrophil/lymphocyte ratios (NLR) in peripheral blood counts at nivolumab treatment were significantly lower in responders (mean of 4.96) than those in non-responders (mean of 10.46) in the group aged < 65 **(C)**, but not in the group aged ≥ 65 **(D)**. **(E–H)** A nivolumab response was significantly associated with high CPS **(E)** and lymphoid-inflamed profiles based on cell densities of nine immune cell lineages **(G)** in the group aged ≥ 65, but not in the group aged < 65 **(F, H)**. Bars and whiskers represent the median and interquartile ranges, respectively. Statistical differences were determined via Kruskal-Wallis tests in **(A–F)** or Chi-square tests in **(G, H)**.

Next, the association between immune characteristics and clinicopathological factors was explored. Focusing on the history of prior therapy related to a favorable response in multivariate analyses (**Table 1**), stratified analysis revealed that the frequency of natural killer (NK) cells was associated with a nivolumab response in patients with prior cetuximab use but not those with a cetuximab-naïve status (**Figures 3A, B**). We then stratified the therapeutic response and related immune characteristics according to patient age. A high NLR was significantly associated with a lower response rate in patients aged < 65 (**Figure 3C**) but not in those aged ≥ 65 (**Figure 3D**). In contrast, CPS showed a significant correlation with a nivolumab response in the group aged ≥ 65 but not in the group aged < 65 (**Figures 3E, F**). When we examined the immune cell composition, we found that lymphoid-inflamed immune profiles were highly associated with a nivolumab response only in patients aged ≥ 65 (**Figures 3G, H**), suggesting that nivolumab-related biomarkers may differ by age.

### Mutational profiles associated with a nivolumab response

**Figure 4:**
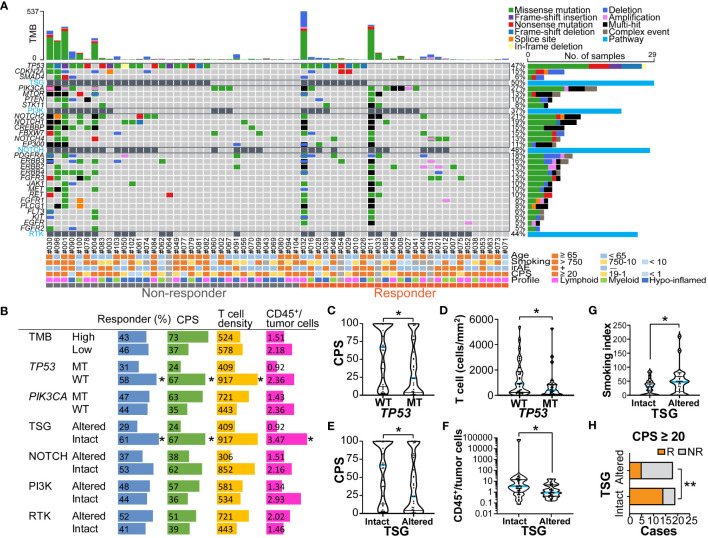
Genomic profiles related to nivolumab outcomes and immune characteristics **(A)** An oncoplot of the top 27 mutated genes is shown. Alterations of cell signaling pathways of tumor suppressor genes (TSG), phosphatidylinositol-3 kinase (PI3K), *NOTCH*, and receptor tyrosine kinases (RTK) were curated by representative genetic alterations (see **Supplementary Table 3**). The upper bar plot indicates the number of intergenic somatic variants per patient while the right bar plot shows the number of variants per gene. Response and baseline characteristics are shown below the oncoplot. **(B)** Responder percentages, and the median values of combined positive score (CPS), total T cell density (cells/mm^2^), and ratios of CD45^+^ immune cells to tumor cells are shown, stratified by tumor mutation burden (TMB) (high ≥ 10mb, and low < 10mb), *TP53* and *PIK3CA* mutations, and pathway alterations. **(C–G)** The violin plots present CPS, T cell density, ratios of CD45^+^ immune cells to tumor cells, and smoking index (pack/year), stratified by *TP53* mutation status **(C, D)**, and TSG pathway alterations **(E, F)**. The bars represent the median and quartile. **(H)** Responders (R) and non-responders (NR) were stratified by the presence or absence of TSG pathway alterations in the group with CPS ≥ 20. Statistical differences were determined via Chi-square tests in **(B, H)** or Kruskal-Wallis tests in **(B-G)**. *p < 0.05, **p < 0.01.

To explore the oncogenic mechanisms related to therapeutic response, genomic DNA was extracted from FFPE specimens and subjected to next-generation sequencing (**Figure 4A** and **Supplementary Figures 3A, B**). No correlation was observed between TMB and a nivolumab response when stratified by 10 mb as the cut-off for TMB (TMB-high, n = 14; TMB-low, n = 48) (**Figure 4B**). Mutations in mismatch repair (MMR) genes were observed in 9/14 (64.3%) TMB-high cases; however, the presence of MMR gene mutations did not correlate with a nivolumab response (5/8 in non-responders; 4/6 in responders). Among the gene mutations analyzed, *TP53* and *PIK3CA* were frequent, with mutation positivity in 46.8% and 29.0% of cases, respectively (**Figure 4A**). Notably, *TP53* mutation was significantly associated with a poor response to nivolumab (**Figure 4B**). There was no correlation between treatment response and *TP53* mutation subtypes, including *TP53* missense or nonsense mutations (**Figure 4A**). *STK11* mutations, known as resistance factors for immunotherapy (18), were observed in 5/62 cases (8.1%), but there was no correlation with responses. Stratification by TMB status did not show that single gene mutations correlated with nivolumab responses (**Supplementary Figures 3A, B**).

To functionally characterize the altered genomic profiles in HNSCC, the set of commonly mutated genes was categorized into representative signaling pathways including the tumor suppressor gene (TSG), PI3 kinase (PI3K) pathway, *NOTCH* pathway, and receptor tyrosine kinase (RTK) pathway (**Figure 4A** and **Supplementary Table 3**). Altered pathways were observed in TSG, *NOTCH*, PI3K, and RTK at rates of 31.3%, 38.7%, 45.5%, and 50.0%, respectively (**Figure 4B**). Interestingly, we observed that altered TSG pathways were significantly more prevalent in the non-responder group than in the responder group (**Figure 4B**), suggesting that TSG mutations might be associated with resistance to nivolumab in HNSCC.

### Associations between mutational and TME profiles in relation to a nivolumab response

The potential associations of genomic and immune profiles and their impact on nivolumab responses were analyzed based on 58 cases in which both could be obtained (**Figure 4B** and **Supplementary Figures 4A, B**). The TMB-high status tended to have higher immune cell densities, TPS and CPS, but the difference was not significant (**Supplementary Figure 5A**). Among the major mutations, the *PIK3CA* mutation-positive group had significantly higher TPS than the wild-type group (**Supplementary Figure 5B**), but not CPS (**Supplementary Figure 5C**), showing a potential association between *PIK3CA* mutation and tumor-intrinsic PD-L1 expression. In contrast, the *TP53* mutation-positive group had significantly lower CPS and T cell densities than the wild-type group (**Figures 4B–D**), and also tended to have lower immune/tumor cell ratios (**Supplementary Figure 5D**), suggesting the presence of immune-excluded microenvironment characteristics. The patients with altered TSG pathway status, including *TP53*, *CDKN2A*, and *SMAD4* mutations, had significantly lower CPS and immune/tumor cell rations, and higher smoking index than those without TSG alterations (**Figures 4B, E–G** and **Supplementary Figure 5E**). These results support our previous observations in which low CPS and high smoking index were associated with poor responses (**Figures 1A**, **2C**). Notably, in the group with CPS ≥ 20, altered TSG pathways were highly correlated with non-responders (**Figure 4H**), indicating that immunotherapy candidates with strong PD-L1 expression may benefit from the ability to anticipate treatment resistance by the presence of TSG mutations. These observations suggest that nivolumab treatment effect was influenced by a potential combination of clinical factors, immune environmental characteristics, and gene mutation profiles in HNSCC.

## Discussion

Although PD-1 antibodies have been widely used in routine clinical practice for R/M HNSCC, there are no clinically available biomarkers other than PD-L1 expression scores; thus, biomarker-guided precision medicine is yet to be realized. This multicenter retrospective study analyzed responders and non-responders based on clinicopathological, immune microenvironmental, and genomic factors. We identified an integrated set of biomarkers associated with nivolumab responses in HNSCC.

Among the clinicopathological factors, HPV-positive cancers had a higher proportion of responders (**Figure 1C**), consistent with previous reports (6), although the interpretation of the results requires caution due to the limitation of having a small number of HPV-positive cases in this study. As was demonstrated in this study (**Supplementary Figure 6**), HPV-positive cancers are known to have a high density of intratumoral CD8^+^ T cells despite T cell exhaustion from possible chronic antigen stimulation, suggesting the potential efficacy of PD-1 inhibitors (19, 20). In this study, a history of alcohol consumption and smoking was associated with poor nivolumab efficacy (**Figures 1A, B**). Because there was no significant difference in drinking and smoking indexes between the HPV-positive cases and the negative/unknown group, HPV status did not appear to be an underlying factor. However, genomic analysis revealed that drinking and smoking levels correlated with an altered TSG pathway, which was associated with a poor response (**Figure 4F**), suggesting the potential involvement of gene mutations related to alcohol and smoking exposure.

Among the TME factors, high PD-L1 expression in tumor and immune cells, CD8^+^ T cell density, and macrophage density were associated with nivolumab responsiveness (**Figure 2**). In the present study, there was a significant correlation between PD-L1 expression and intratumoral CD8^+^ T cell density (**Figure 2F**). Given that PD-L1 expression is triggered by inflammatory cytokines, such as interferon-γ, high PD-L1 status potentially reflects a T cell-mediated inflammatory status (21). Interestingly, abundant macrophage density was associated with this response (**Figure 2D**). Although most tumor-associated macrophages possess cancer-promoting M2-like properties (8), the macrophages associated with the therapeutic effect observed in this study may be antigen-presenting M1-like populations. However, fractional analysis was not possible in this study. Together, TME profiles associated with a poor response to immunotherapy are related to cold tumor status, characterized by low immune cell density and PD-L1 expression, which requires additional strategies to convert cold to hot tumors (22).

Using peripheral blood data and tumor tissue profiles stratified by patient age and prior treatment may provide better predictive biomarkers for nivolumab responses in HNSCC (**Figure 3**). First, among patients with a history of cetuximab use, those with a high density of NK cells within the tumor showed a favorable response to nivolumab, suggesting a possible correlation with the antibody-dependent cell-mediated cytotoxicity (**Figures 3A, B**). Second, the association between response and PD-L1 expression was stronger in patients aged ≥ 65, while some patients under 65 showed CR, even with low PD-L1 levels (**Figures 3E, F**). Since it is well recognized that immunological senescence causes quantitative and/or qualitative alterations in immune cells (23), the potential mechanisms of PD-1 treatment resistance might differ by aging-induced immunological backgrounds. Those observations suggest that biomarkers can be personalized depending on the clinicopathological background and treatment sequence.

In this study, *TP53* mutations were associated with immune-exclusionary microenvironment and impaired immunotherapy response in HNSCC (**Figures 4B, D**). *TP53* mutations are the most common genetic alterations, occurring in 30–80% of HNSCC, which are related to increased cell proliferation, metastatic potential, skewed metabolism, and immune escape (24, 25). Our genomic analysis also demonstrated a lack of association between the TMB and treatment effects. Compared to other cancers, HNSCC is known to have a low correlation between TMB and T cell-inflamed signature (12), which is compatible with our results. These results corroborate the previous notion that *TP53* mutations, which are often linked to high TMB, may compromise the prognostic value of TMB for immunotherapy outcomes (26). In particular, the existence of tumor suppressor gene alterations mainly *TP53* was shown to identify non-responders among PD-L1 high expression subjects (**Figure 4G**), suggesting that mutational profiles could be used to stratify PD-L1-high immunotherapeutic candidates. These findings suggest that immunotherapeutic candidates with *TP53* mutation might require careful monitoring, including a prompt transition to second-line treatment when necessary. This nuanced approach could assist in patient selection and management, ensuring that all patients, including those with *TP53* mutations, have the best chance of benefiting from immunotherapy. Furthermore, the involvement of epigenomics and gene expression, including *MYC* amplification, in the therapeutic effect of immunotherapy has been suggested (27), and further analysis of transcriptomics and epigenomics, in addition to proteomics and genomics analyzed in this study, may help identify the optimal therapeutic group.

One notable limitation of our investigation is the absence of survival data, which precludes a direct correlation between the identified biomarkers and long-term clinical benefits such as progression free and overall survival. Another limitation of this study is the insufficient number of cases, which precluded sub-group analyses based on HPV status or primary sites, and the creation of a nomogram integrating the identified prognostic factors. A critical limitation of our study is the inherent constraints of our comprehensive case collection approach, which precluded the possibility of conducting *a priori* sample size calculation or case-control matching. This methodological choice inevitably introduces a notable degree of heterogeneity within the study population, although the clinical characteristics of the responder and non-responder groups were largely aligned. In TMB analyses, while our uracil-DNA glycosylase treatment was aimed at reducing C:G > T:A variant artifacts, we cannot completely rule out the possibility of residual artifacts, especially in cases with extremely high TMB. Therefore, caution is required when interpreting these results. These highlight the necessity for larger-scale prospective studies to enable comprehensive stratification and predictive modeling, which could significantly enhance the clinical utility of these biomarkers.

This study demonstrates the possibility of using integrative biomarkers based on clinicopathological factors, TME, and genomic characteristics. All biomarkers identified in this study, except immune-related adverse events, could be obtained prior to treatment and might be useful in predicting the therapeutic effect of nivolumab for R/M HNSCC. Furthermore, the immunotherapeutic resistance features of low PD-L1 related to *TP53* mutations and cold immune microenvironmental profiles found in this study could aid in the development of optimized combination strategies, including chemo- and targeted therapies, to maximize the effects of cancer immunotherapy in HNSCC.

## Data availability statement

The original contributions presented in the study are included in the article/**Supplementary Material**. Further inquiries can be directed to the corresponding author.

## Ethics statement

The studies involving humans were approved by the Institutional Review Board (Tokyo Medical and Dental University: G2018-009-02). The studies were conducted in accordance with the local legislation and institutional requirements. The participants provided their written informed consent to participate in this study.

## Author contributions

TT: Writing – review & editing, Writing – original draft, Visualization, Validation, Resources, Methodology, Investigation, Funding acquisition, Formal analysis, Data curation, Conceptualization. KO: Resources, Methodology, Writing – review & editing, Writing – original draft, Visualization, Validation, Investigation, Funding acquisition, Formal analysis, Data curation, Conceptualization. KM: Resources, Funding acquisition, Writing – review & editing, Writing – original draft, Visualization, Validation, Methodology, Investigation, Formal analysis, Data curation, Conceptualization. SSa: Writing – review & editing, Data curation. JM: Writing – review & editing, Data curation. KY: Supervision, Project administration, Funding acquisition, Conceptualization, Writing – review & editing. AK: Writing – review & editing, Data curation. HM: Writing – review & editing, Data curation. HOg: Resources, Methodology, Writing – review & editing, Software. SSh: Resources, Methodology, Writing – review & editing, Software. TAk: Writing – review & editing, Methodology. MK: Writing – review & editing, Methodology. II: Writing – review & editing, Methodology. YaS: Writing – review & editing, Resources. SK: Writing – review & editing, Resources. AW: Writing – review & editing, Resources. TY: Writing – review & editing, Resources. YA: Writing – review & editing, Resources. RH: Writing – review & editing, Resources. YuS: Writing – review & editing, Resources. HOz: Writing – review & editing, Resources. KT: Writing – review & editing, Resources. NO: Writing – review & editing, Resources. DS: Writing – review & editing, Resources. AH: Writing – review & editing, Resources. YU: Writing – review & editing, Resources. TM: Writing – review & editing, Resources. NM: Writing – review & editing, Resources. NH: Writing – review & editing, Resources. TFukusumi: Writing – review & editing, Resources. HI: Writing – review & editing, Resources. TakuoF: Writing – review & editing, Resources. TFujii: Writing – review & editing, Resources. KN: Writing – review & editing, Resources. SI: Writing – review & editing, Resources. TU: Writing – review & editing, Resources. NC: Writing – review & editing, Resources. RY: Writing – review & editing, Resources. MiM: Writing – review & editing, Resources. HU: Writing – review & editing, Resources. TO: Writing – review & editing, Resources. MuM: Resources, Writing – review & editing. ST: Writing – review & editing, Resources. KI: Writing – review & editing, Software, Resources, Methodology, Funding acquisition. SH: Writing – review & editing, Supervision, Resources, Funding acquisition. TAs: Writing – review & editing.
