# Supplementary material for: Clinical, genomic and immune microenvironmental determinants of nivolumab response in head and neck squamous cell carcinoma
Source: Front Immunol. 2024 Jul 29;15:1390873. doi: 10.3389/fimmu.2024.1390873 (PMC11317249; doi:10.3389/fimmu.2024.1390873)
Supplement: Supplementary file 1 [file DataSheet_1.docx]

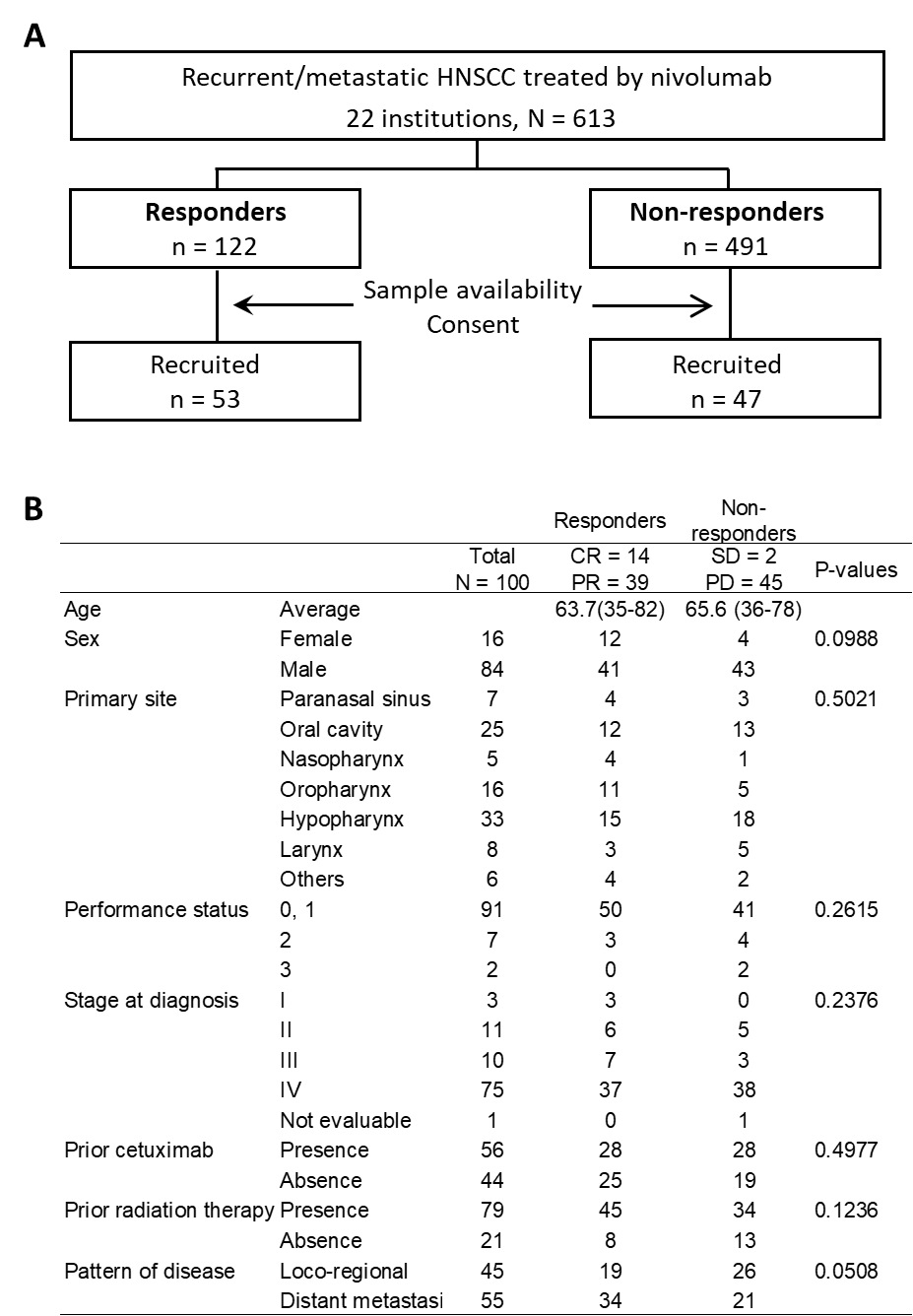


**Supplementary Fig. 1. Study diagram and clinicopathological factors.** (**A**) This study encompassed patients with recurrent/metastatic head and neck squamous cell carcinoma who underwent nivolumab treatment across 22 facilities from March 1, 2017, to May 31, 2018. From a total of 613 cases, the study incorporated 53 responders and 47 non-responders in a case-control design. (**B**) The figure presents the clinicopathological information. Statistical significance was determined by chi-square tests.

**
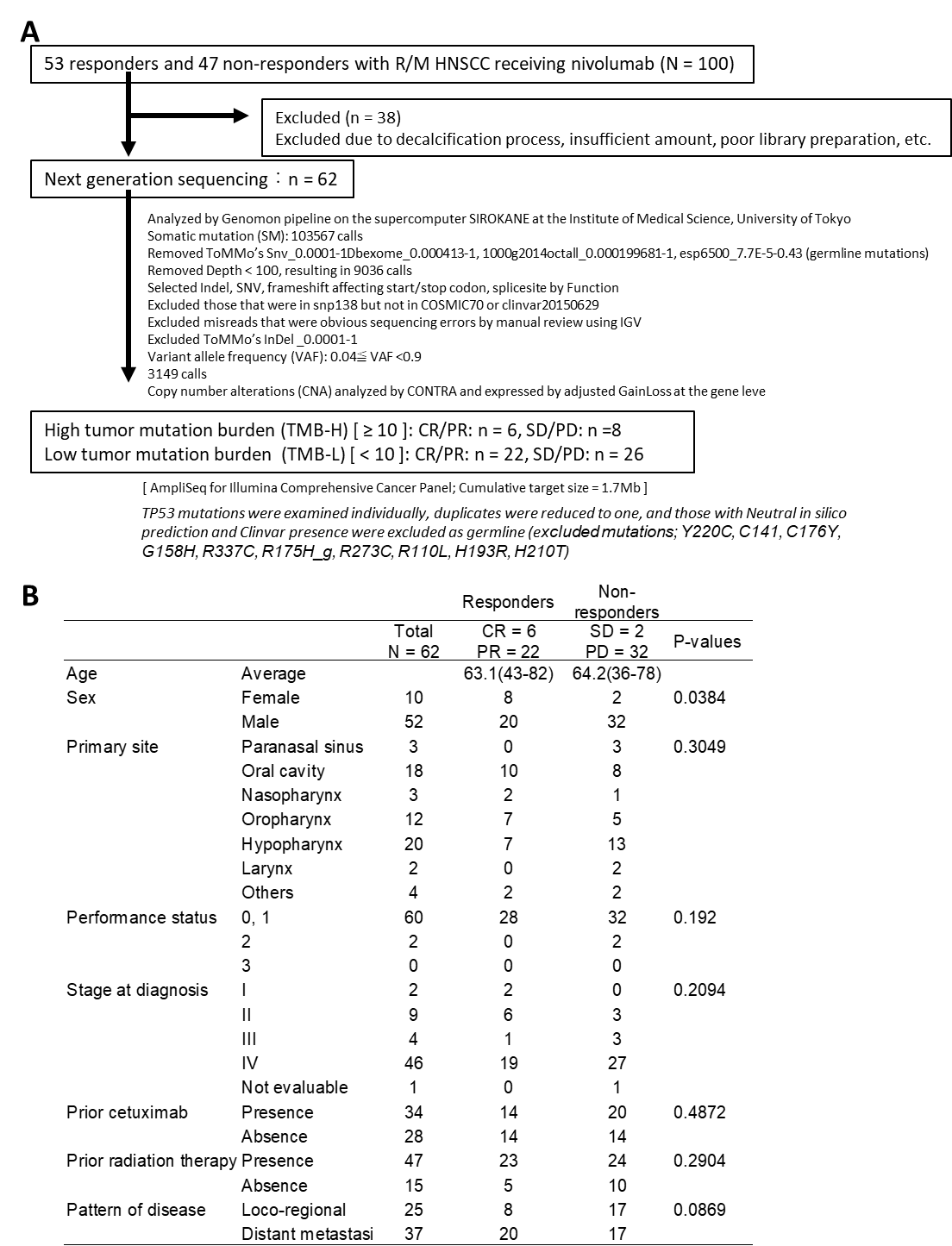
****Supplementary Fig 2. Study diagram and clinicopathological factors in gene mutational analysis.** (**A**) A flowchart of the gene mutation validation process in this study. (**B**) The figure presents the clinicopathological information in the cases of gene mutation analysis (n = 62). Statistical significance was determined by chi-square tests.

**
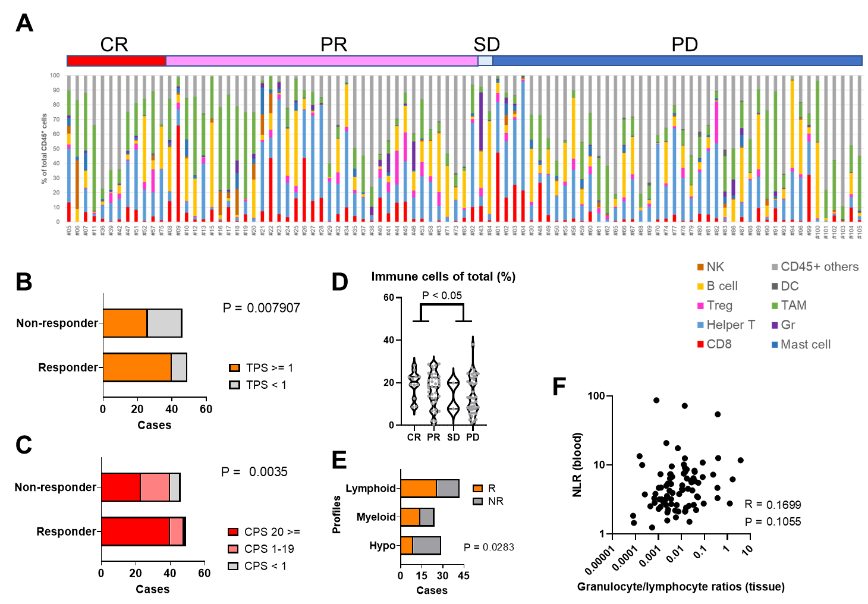
**

**Supplementary Fig. 3. Multiplex immunohitochemistry-based immune profiling in support of Fig. 2.**

(**A**) Immune cell composition is shown comparing therapeutic responses. (**B**) Tumor proportion score (TPS) comparing responders and non-responders. (**C**) Combined positive score (CPS) comparing responders and non-responders. (**D**) Cell percentages of CD45^+^ immune cells in total intratumoral cells were shown, comparing different therapeutic responses. (**E**) Responders and non-responders were shown by immune profiles. (**F**) Spearman correlations of intratumoral granulocyte/lymphocyte ratios versus circulating neutrophil/lymphocyte ratios (NLR) were shown. Statistical differences were determined via Chi-square tests in (B), (C), and (E) or Kruskal-Wallis tests in (D).

**
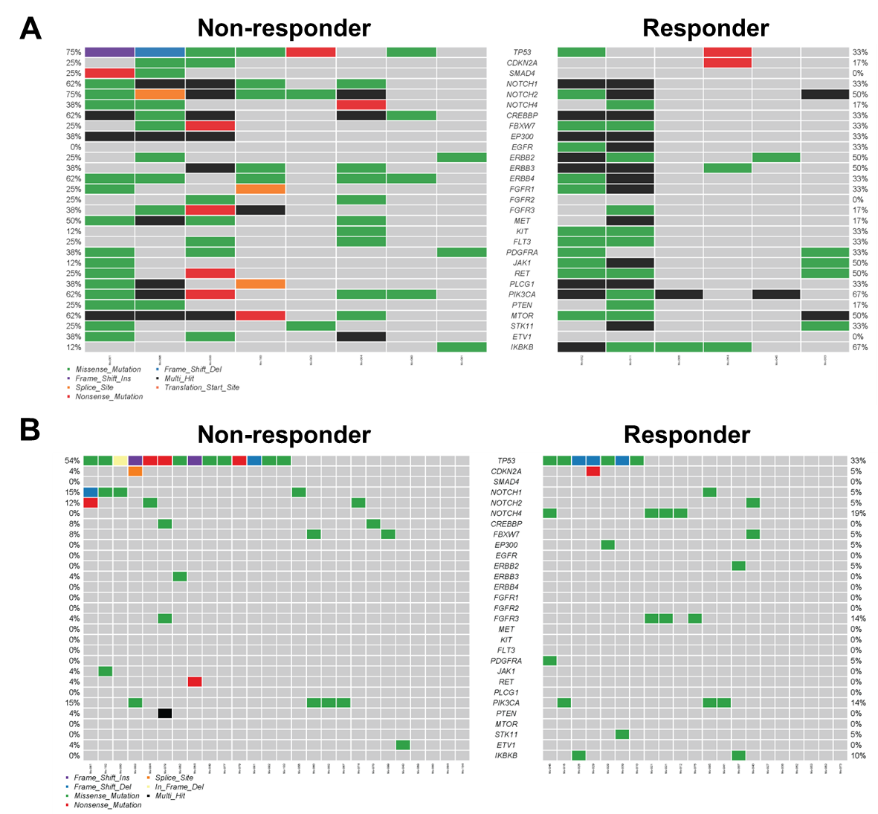
**

**Supplementary Fig. 4. Oncoplots of gene mutations stratified by tumor mutation burden (TMB) in support of Fig. 4.**

(**A-B**) Oncoplots of frequent gene mutations were shown in the subgroups of TMB-high (A) and TMB-low (B).

**­**
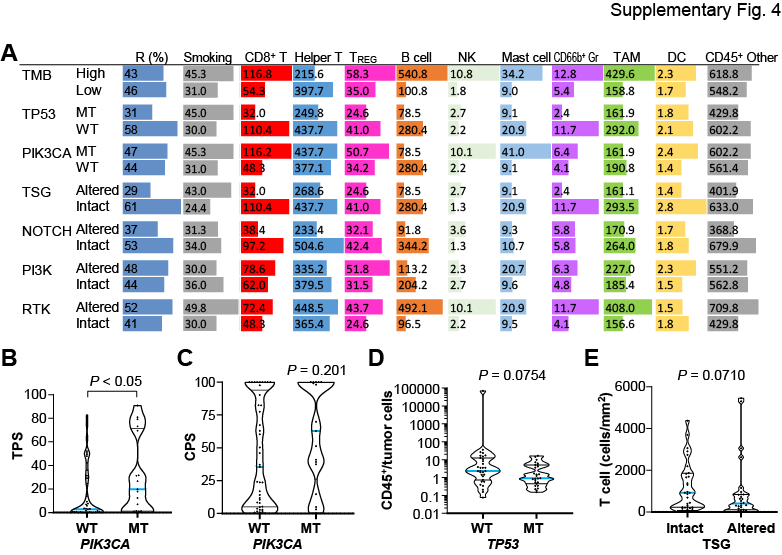


**Supplementary Fig. 5. Immune-related parameters in relation to genomic profiles in support of Fig. 4.**

(**A**) Responder percentages, and median values of smoking index (pack/year), tumor proportional score (TPS), immune cell densities (cells/mm2), and ratios of CD45^+^ immune cells to tumor cells are shown, comparing the status of tumor mutation burden (TMB) (high ≥ 10mb, and low < 10mb), TP53 and PIK3CA mutations, and pathway alternations. (**B-C**) The violin plots compare tumor proportion scores (TPS) (B), and combined positive scores (CPS) (C) by *PIK3CA* mutation status. (**D**) The violin plots compare ratios of CD45^+^ immune cells to tumor cells, stratified by *TP53* mutation status. (**E**) The violin plots compare T cell density, stratified by TSG (tumor suppressor genes) pathway status. Bars represent median, and interquartile range. Statistical differences were determined via Kruskal-Wallis tests.

**Supplementary Fig. 6. Immune cell densities and human papilloma virus (HPV) status.** Immune cell densities (cells/mm^2^) of CD8^+^ T cells (CD8), helper T cells, regulatory T cells (Treg), B cells, natural killer cells (NK), mast cells, CD66b^+^ granulocytes (Gr), tumor associated macrophages (TAM), and dendritic cells (DC) were quantified, comparing p16 negative/unknown and p16 positive cases. Bars and boxes/whiskers represent the median and interquartile ranges, respectively. Statistical differences were determined via Kruskal-Wallis tests.

**Supplementary Table 1.** The list of antibodies and conditions used for staining.

**Supplementary Table 2.** A list of lineage identification markers.

**Supplementary Table 3.** The list of pathway alterations curated by gene mutations
